# Is marital status associated with quality of life?

**DOI:** 10.1186/s12955-014-0109-0

**Published:** 2014-08-08

**Authors:** Kyu-Tae Han, Eun-Cheol Park, Jae-Hyun Kim, Sun Jung Kim, Sohee Park

**Affiliations:** Department of Public Health, Graduate School, Yonsei University, Seoul, Republic of Korea; Institute of Health Services Research, Yonsei University College of Medicine, Seoul, Republic of Korea; Department of Preventive Medicine, Yonsei University College of Medicine, Seoul, Republic of Korea; Department of Biostatistics, Graduate School of Public Health, Yonsei University, 50 Yonsei-ro, Seodaemun-gu, Seoul, 120-752 Republic of Korea

**Keywords:** EQ-VAS, EQ-5D, Marital status, Quality of life

## Abstract

**Background:**

The divorce rate has been increasing rapidly in Korea; the single rate and trends in divorce are also changing rapidly. This study aimed to examine the relationship between marital status and quality of life (QOL) in an attempt to understand these changes. We also investigated the relationship between QOL and marital status by age group.

**Methods:**

We used data from the Community Health Survey (2008: *n* = 200,800; 2009: *n* = 227,700; 2010: *n* = 229,229) administered by the Korean Centers for Disease Control and Prevention. After excluding 63,527 participants with incomplete information on QOL and/or marital status, the final analysis involved 594,202 participants. The analysis used *t*-tests and Chi-square tests to compare demographic variables between men and women, and ANOVA to compare QOL scores among comparison groups. We also performed a multilevel analysis on the relationship between QOL and marital status while accounting for the provincial differences.

**Results:**

The multilevel analysis by marital status showed that single men had significantly worse QOL (both EQ-VAS and EQ-5D) than married men. On the other hand, the QOL measured by EQ-VAS was better in single women than in married, and separated or divorced women. When QOL was assessed using EQ-5D, single and separated or divorced women had worse scores than married women. In the analysis by age group, the QOL of married men under the age of 30 years was lower than that of single men or men with marriage problems as measured by EQ-VAS. However, among 40–69-year-old men, married men had the highest QOL values. Similarly, for women in their 30s, single women had the highest EQ-VAS values, but for 40–69-year-old women, single women had lower EQ-VAS scores than married women.

**Conclusion:**

There was significant relationship between marital status and QOL, and this relationship appeared to differ by gender and age.

## Background

Life Satisfaction as measured by the Better Life Index is an indicator of quality of life (QOL) and is calculated based on 11 indicators. In a report issued by the OECD in 2013, among the 37 countries examined, South Korea held a low position, 27^th^ place (Korea’s score: 6.0; OECD average: 6.6/10). It is apparent that life satisfaction among Koreans is low, even in comparisons using other indicators [1], no definitive factors have been identified that explain why life satisfaction or QOL is so low. Previous studies to clarify factors affecting QOL have considered relevant socio-economic conditions and socioeconomic status generally. However, few studies have examined how marriage status impacts QOL [2–4].

Marital status has been classified as single, married, and marriage problems (including separation, divorce, and bereavement). The situation for each group in this classification in South Korea is as follows. According to the 2008 OECD Family Database, the divorce rate in Korea is at a high level relative to other OECD countries (Korea: 2.6; OECD average: 2.1 of 7), and the divorce rate increased rapidly from 1970 to 2008 (change from 1970 to 2008: 2.2/4 total points) [5]. Moreover, according to the Population Trends Survey of the Korean National Statistical Office in 2000–2012, the divorce rate for men over 40 increased from 46.5% in 2000 to 70.5% in 2012, and the divorce rate for women increased from 54.2% to 74.5%. These numbers indicate that trends in divorce rates are changing [6]. According to the Population and Housing Census department of Statistics Korea, the single rate among men 25–39 years old rose from 30.0% in 1995 to 52.8% in 2010. Similarly, the single rate among 25–39-year-old women rose from 13.2% to 35.6% during the same period [7]. Thus, given that the trends in marital status among the Koreans are changing rapidly, a study on whether sudden changes in marital status influence QOL is needed.

Of course, there have been previous studies of QOL related to marital status. However, those studies focused on topics such as the social role of the spouse in mental health, the impact of marital status on a particular disease, or the impact of marital status in preventing certain diseases [8–15]. However, reported research on the impact of the rapid changes in marital status and on the relationship between marital status and QOL in each age group is lacking. Thus, in this study, we analyzed the differences in QOL by marital status and examined the relationship of marital status with QOL according to sex and age group.

## Methods

### Study population

The data used were from the Community Health Survey administered by the Korean Centers for Disease Control and Prevention, which was designed to facilitate inter-provincial comparisons [16]. The Community Health Survey was administered by investigators who conducted one-on-one visits and interviews targeting adults 19 years of age or older in 253 health centers nationwide starting in 2008. Data were gathered for 200,800 people in 2008, 227,700 people in 2009, and 229,229 people in 2010. These were integrated and sampling weights were incorporated for the analysis. The final analysis used data from 594,202 people from the total of 657,729 after excluding 63,527 people for whom information on QOL and/or marital status was incomplete and therefore could not be analyzed. Because the Community Health Survey data is a secondary data that do not contain private information and is available to public domain, our study did not have to address ethical concerns.

### Variables

The outcome variables were scores on the EQ-VAS and EQ-5D Index. EQ-VAS is a self-rated health questionnaire presented as a vertical visual analog scale, where the endpoints are labeled “best” and “worst imaginable health state”. Participants completed the scale ranging from 0 to 100 on the study day. Responses to this scale were used as a quantitative measure of participants’ self-rated health. The EQ-5D is an index of five dimensions of health-related QOL. The five dimensions are mobility, self-care, usual activities, pain/discomfort, and anxiety/depression. Original EQ-5D index has values ranging from 0 to 1. For the purpose of comparing the two indicators (EQ-VAS and EQ-5D), the EQ-5D Index was multiplied by 100 before the data were analyzed.

The variable of major interest in its association with the outcome variables was marital status. Marital status was divided into married, single, and marriage problems (separation, divorce, bereavement). Other independent variables considered in the analysis were frequent depression for more than 2 weeks, awareness of stress, age, family income, education level, perceived health status, and survey year. Stress awareness was defined as the endorsing “a lot” or “very much” as descriptive of stress in one’s daily life. Age was classified into 5-years intervals. Family income was classified into four groups. Education levels were classified as “less than high school”, “high school education,” and “university education.” Subjective health status was defined describing one’s subjective health level as “good” or “very good”.

The provincial variables in the analysis reflected the characteristics of the Community Health Survey data used in the analysis. Provincial variables were based on the e-provincial indicators of the survey conducted by Statistics Korea by year, as follows: resident population, gross provincial domestic product (GRDP), crude divorce rate, and married couple [17]. The e-provincial indicators were variables representing the 16 provinces. GRDP as value-added on the production side was used as an indicator to measure how much value added to economic activities in each region.

### Statistical analysis

For comparisons related to QOL, we analyzed men and women separately. For the analysis of the relationship between QOL and marital status, the following variables were adjusted: frequent depression for more than 2 weeks, stress awareness, age, family income, education level, perceived health status, survey year, and provincial variables. To compare the relationship between QOL and marital status by age group, age was divided into 5-year intervals. We first examined the distribution of each variable to analyze the general characteristics of each group, and we performed *t*-tests and χ^2^ tests to examine differences in each variable according to gender. Next, to compare the average values on the QOL indices according to the independent variables, we performed analyses of variance (ANOVAs). Finally, to analyze the relationship between QOL and marital status, considering the characteristics of the Community Health Survey, we performed a multilevel analysis. All analyses were performed using SAS software (ver. 9.2). *P*-values <0.05 were considered to indicate statistical significance.

## Results

Of the 594,202 participants in the final sample, 46.0% were men, and 54.0% were women. A higher proportion of married persons were in men than in women. Women were almost twice as likely as were men to report frequent depression for more than 2 weeks, and awareness of stress was higher in women than for men. The subjective health status of men was higher than that of women. Regarding provincial variables, the resident registration population of men was slightly higher than that of women, and the average of GRDP was higher in men than in women (Table 1).

**Table 1:** **Characteristics of study participants (frequency, %)**

	**Total**	**Men**	**Women**	***P*** -value**
	594202	273537	320665	
**Marital status**				
Single	88345	50625 (18.5)	37720 (11.8)	<.0001
Separation/Divorced/Bereavement	99978	23189 (8.5)	76789 (23.9)	
Married	405879	199723 (73.0)	206156 (64.3)	
**Frequent depression for more than 2 weeks**				
Yes	42751	13566 (5.0)	29185 (9.1)	<.0001
No	551451	259971 (95.0)	291480 (90.9)	
**Stress awareness**				
Yes	157857	72630 (26.6)	85227 (26.6)	0.8208
No	436345	200907 (73.4)	235438 (73.4)	
**Age (years)**				
19–24	33074	14950 (5.5)	18124 (5.7)	<.0001
25–29	41418	19842 (7.3)	21576 (6.7)	
30–34	47728	22592 (8.3)	25136 (7.8)	
35–39	60048	29041 (10.6)	31007 (9.7)	
40–44	59140	28984 (10.6)	30156 (9.4)	
45–49	62147	30098 (11.0)	32049 (10.0)	
50–54	59263	28178 (10.3)	31085 (9.7)	
55–59	48223	22464 (8.2)	25759 (8.0)	
60–64	45010	20909 (7.6)	24101 (7.5)	
65–69	48444	21340 (7.8)	27104 (8.5)	
70–74	42405	18059 (6.6)	24346 (7.6)	
≥75	47302	17080 (6.2)	30222 (9.4)	
**Family income (thousands won)**				
≤12000	168024	68474 (25.0)	99550 (31.0)	<.0001
12000–24000	142130	68491 (25.0)	73639 (23.0)	
24000–42000	151510	73286 (26.8)	78224 (24.4)	
≥42000	132538	63286 (23.2)	69252 (21.6)	
**Education**				
Less than high school	242564	87985 (32.2)	154579 (48.2)	<.0001
High school graduate	203879	104923 (38.4)	98956 (30.9)	
College graduate	147759	80629 (29.5)	67130 (20.9)	
**Perceived health status**				
Good	247659	129014 (47.2)	118645 (37.0)	<.0001
Bad	346543	144523 (52.8)	202020 (63.0)	
**Year of survey**				
2008	176919	81547 (29.8)	95372 (29.7)	0.0554
2009	210588	97326 (35.6)	113262 (35.3)	
2010	206695	94664 (34.6)	112031 (34.9)	
**Provincial variables**				
Resident population*		4613502 (3973307)	4603006 (3953958)	0.3089
Gross provincial domestic product (GRDP, million won)*		99153215 (82449380)	99145655 (82397934)	0.9719
Crude divorce rate*		2.3 (0.2)	2.3 (0.2)	<.0001
Married (couple)*		29482.1 (27,431.7)	29400.6 (27,317.1)	0.2529

*shown as Mean, SD for all 16 provinces.

**P-values are for results of χ^2^ tests for categorical variables and independent *t*-tests for continuous variables.

The overall ANOVA revealed that the average EQ-VAS score was higher for men than for women. In terms of the relationship between QOL and marital status, men and women had similar results. EQ-VAS scores were higher in the order single > married > marriage problems for both men and women. The overall QOL, measured by the EQ-5D Index, was higher for men than women. The QOL measured by the EQ-5D Index was higher in the order single > married > the marriage problems in both men and women (Table 2).

**Table 2:** **Relationships of quality of life with demographic characteristics and health behaviors [mean (SD) and** ***p*** **-values]**

	**Men**				**Women**			
	**EQ-VAS**		**EQ-5D**		**EQ-VAS**		**EQ-5D**	
**Marital status**								
Single	78.8 (14.6)	<.0001	97.9 (8.2)	<.0001	77.7 (14.4)	<.0001	97.9 (7.1)	<.0001
Separation/Divorced/Bereavement	71.6 (17.6)		92.3 (15.0)		65.2 (18.7)		85.0 (18.5)	
Married	74.9 (15.7)		95.3 (12.3)		72.9 (16.2)		94.2 (11.7)	
**Frequent depression for more than 2 weeks**								
Yes	62.2 (21.8)	<.0001	82.4 (25.3)	<.0001	60.7 (20.6)	<.0001	82.2 (21.5)	<.0001
No	76.0 (15.1)		96.2 (10.4)		72.7 (16.3)		93.4 (12.5)	
**Stress awareness**								
Yes	70.9 (17.6)	<.0001	93.5 (15.7)	<.0001	65.5 (18.9)	<.0001	88.5 (17.7)	<.0001
No	77.0 (14.8)		96.3 (10.3)		73.9 (15.8)		93.8 (12.0)	
**Age (years)**								
19–24	81.8 (13.5)	<.0001	98.9 (5.0)	<.0001	78.8 (14.2)	<.0001	98.7 (4.5)	<.0001
25–29	79.8 (13.6)		98.9 (5.1)		77.3 (14.0)		98.4 (5.0)	
30–34	77.8 (13.5)		98.7 (5.5)		76.5 (14.0)		98.2 (5.3)	
35–39	77.3 (13.6)		98.4 (6.0)		76.8 (13.9)		98.0 (5.8)	
40–44	77.3 (13.8)		98.1 (6.7)		76.3 (14.2)		97.5 (6.8)	
45–49	77.1 (14.3)		97.5 (8.2)		75.3 (14.9)		96.6 (8.0)	
50–54	76.5 (14.9)		96.9 (9.0)		73.5 (15.6)		95.1 (9.8)	
55–59	75.5 (15.4)		96.0 (10.5)		71.5 (16.1)		93.3 (11.4)	
60–64	74.0 (16.1)		94.6 (12.3)		68.8 (16.9)		90.2 (13.6)	
65–69	71.2 (17.2)		91.7 (15.5)		65.3 (17.9)		86.0 (16.1)	
70–74	68.4 (18.2)		88.6 (17.8)		62.2 (18.3)		82.4 (17.7)	
≥75	63.9 (19.5)		81.9 (23.0)		59.2 (19.2)		76.4 (21.9)	
**Family income (thousands won)**								
≤12,000	69.2 (18.7)	<.0001	89.6 (18.0)	<.0001	65.0 (18.8)	<.0001	85.7 (17.7)	<.0001
12,000–24,000	75.8 (15.2)		96.4 (10.3)		72.6 (16.2)		94.0 (12.2)	
24,000–42,000	77.7 (13.8)		97.9 (7.5)		75.0 (14.9)		95.9 (9.9)	
>42,000	78.9 (13.2)		98.3 (6.5)		76.4 (14.5)		96.5 (9.2)	
**Education**								
Less than high school	70.3 (17.9)	<.0001	90.8 (16.8)	<.0001	66.3 (18.3)	<.0001	86.9 (17.2)	<.0001
High school graduate	77.0 (14.8)		97.2 (9.0)		75.9 (14.7)		97.1 (7.4)	
College graduate	78.7 (13.1)		98.4 (6.3)		77.7 (13.3)		98.2 (5.3)	
**Perceived health status**								
Good	82.0 (11.8)	<.0001	98.9 (4.7)	<.0001	80.5 (12.3)	<.0001	98.1 (5.8)	<.0001
Bad	69.5 (16.6)		92.5 (15.3)		66.4 (17.3)		89.0 (16.1)	
**Years**								
2008	75.5 (16.4)	<.0001	95.1 (12.8)	<.0001	71.6 (17.6)	<.0001	92.3 (14.3)	<.0001
2009	75.6 (15.6)		95.8 (11.6)		71.8 (16.9)		92.7 (13.8)	
2010	74.9 (15.5)		95.6 (11.7)		71.5 (16.8)		92.3 (13.8)	
**Total**	75.4 (15.8)	<.0001	95.5 (12.0)	<.0001	71.6 (17.1)	<.0001	92.4 (14.0)	<.0001

*P-values for results by ANOVA.

A multilevel analysis of the EQ-VAS was conducted to investigate the relationship between QOL and marital status while adjusting for potential confounding variables such as age, depression, stress, and socioeconomic status. Based on marital status, men had higher EQ-VAS values in the order married > single > marriage problems, and women had higher values in the order single > married > marriage problems (male single: −0.567, separation/divorce/bereavement: −0.966; female single: 0.760, separation/divorce/bereavement: −0.544; *p* < 0.05). The multilevel analysis of EQ-5D by marital status revealed that men had higher values in the order married > marriage problems > single. EQ-5D values for women were higher in the order married > single > marriage problems (male single: −0.904, separation/divorce/bereavement: −0.707; female single: -0.273, separation/divorce/bereavement: −0.822; *p* < 0.05; Table 3).

**Table 3:** **Multilevel analysis results of EQ-VAS and EQ-5D (estimated regression coefficient,** ***P*** -value*)

	**Men**				**Women**			
	**EQ-VAS**		**EQ-5D**		**EQ-VAS**		**EQ-5D**	
**Marital status**								
Single	−0.567	<.0001	−0.904	<.0001	0.760	<.0001	−0.273	<.0001
Separation/Divorced/Bereavement	−0.966	<.0001	−0.707	<.0001	−0.544	<.0001	−0.822	<.0001
Married	-		-		-		-	
**Frequent depression for more than 2 weeks**								
Yes	−7.242	<.0001	−8.545	<.0001	−6.164	<.0001	−6.783	<.0001
No	-		-		-		-	
**Stress awareness**								
Yes	−4.424	<.0001	−1.654	<.0001	−5.914	<.0001	−2.998	<.0001
No	-		-		-		-	
**Age (years)**								
19–24	-		-		-		-	
25–29	−1.630	<.0001	−0.118	0.1215	−0.712	<.0001	−0.202	0.0172
30–34	−2.684	<.0001	−0.375	<.0001	−0.718	<.0001	−0.410	<.0001
35–39	−2.597	<.0001	−0.480	<.0001	−0.077	0.5818	−0.542	<.0001
40–44	−2.363	<.0001	−0.675	<.0001	0.127	0.3804	−0.618	<.0001
45–49	−2.030	<.0001	−1.000	<.0001	0.371	0.0107	−0.844	<.0001
50–54	−1.891	<.0001	−1.143	<.0001	−0.276	0.0699	−1.409	<.0001
55–59	−2.232	<.0001	−1.651	<.0001	−0.577	0.0004	−2.293	<.0001
60–64	−2.692	<.0001	−2.522	<.0001	−1.958	<.0001	−4.442	<.0001
65–69	−3.815	<.0001	−4.140	<.0001	−3.887	<.0001	−7.695	<.0001
70–74	−5.303	<.0001	−6.656	<.0001	−6.300	<.0001	−11.378	<.0001
≥75	−8.989	<.0001	−12.833	<.0001	−9.805	<.0001	−17.716	<.0001
**Family income (thousands won)**								
≤12,000	−3.599	<.0001	−2.830	<.0001	−3.009	<.0001	−1.943	<.0001
12,000–24,000	−1.245	<.0001	−0.329	<.0001	−1.313	<.0001	−0.243	<.0001
24,000–42,000	−0.660	<.0001	−0.033	0.4518	−0.602	<.0001	−0.078	0.0948
>42,000	-		-		-		-	
**Education**								
Less than high school	−2.609	<.0001	−2.089	<.0001	−2.375	<.0001	−2.088	<.0001
High school graduate	−0.958	<.0001	−0.382	<.0001	−0.627	<.0001	−0.210	<.0001
College graduate	-		-		-		-	
**Perceived health status**								
Good	9.323	<.0001	2.517	<.0001	9.067	<.0001	2.792	<.0001
Bad	-		-		-		-	
**Years**								
2008	-		-		-		-	
2009	−0.549	<.0001	0.102	0.217	−0.461	0.0005	−0.298	0.0015
2010	−1.374	<.0001	−0.105	0.0877	−0.744	<.0001	−0.427	<.0001
**Provincial variables (per 100,000 people)**								
Resident population	−0.071	<.0001	0.030	<.0001	−0.066	<.0001	0.092	<.0001
Gross provincial domestic product (GRDP, million won)	−0.001	<.0001	−0.001	<.0001	−0.001	<.0001	−0.003	<.0001
Crude divorce rate	98030.000	0.0219	33110.000	0.1671	5775.000	0.8952	6963.000	0.8241
Married (couple)	12.000	0.0003	−2.000	0.2347	12.900	<.0001	−5.000	0.0225

*P-values for results of multilevel analysis.

When examined in detail the relationship between QOL and marital status by sex and age group, EQ-VAS scores in men younger than 30 years old who were single or had marriage problems indicated better QOL than was found in married men (age 19–24, single: 2.028, *p* < 0.05; separation/divorce/bereavement: 0.2906, *p* > 0.05; age 25–29, single: 0.977, *p* < 0.05; separation/divorce/bereavement: 1.894, *p* < 0.05). In the 35–69-year age group among men, single men tended to have lower QOL scores than did married men. In the 40–64-year interval, the estimated QOL score was higher in the order married > marriage problems and single. In the analysis of the EQ-5D, there was a slightly different tendency from that seen for EQ-VAS. Regardless of age, in men, QOL was in the order married > marriage problems and single.

**Figure 1:**
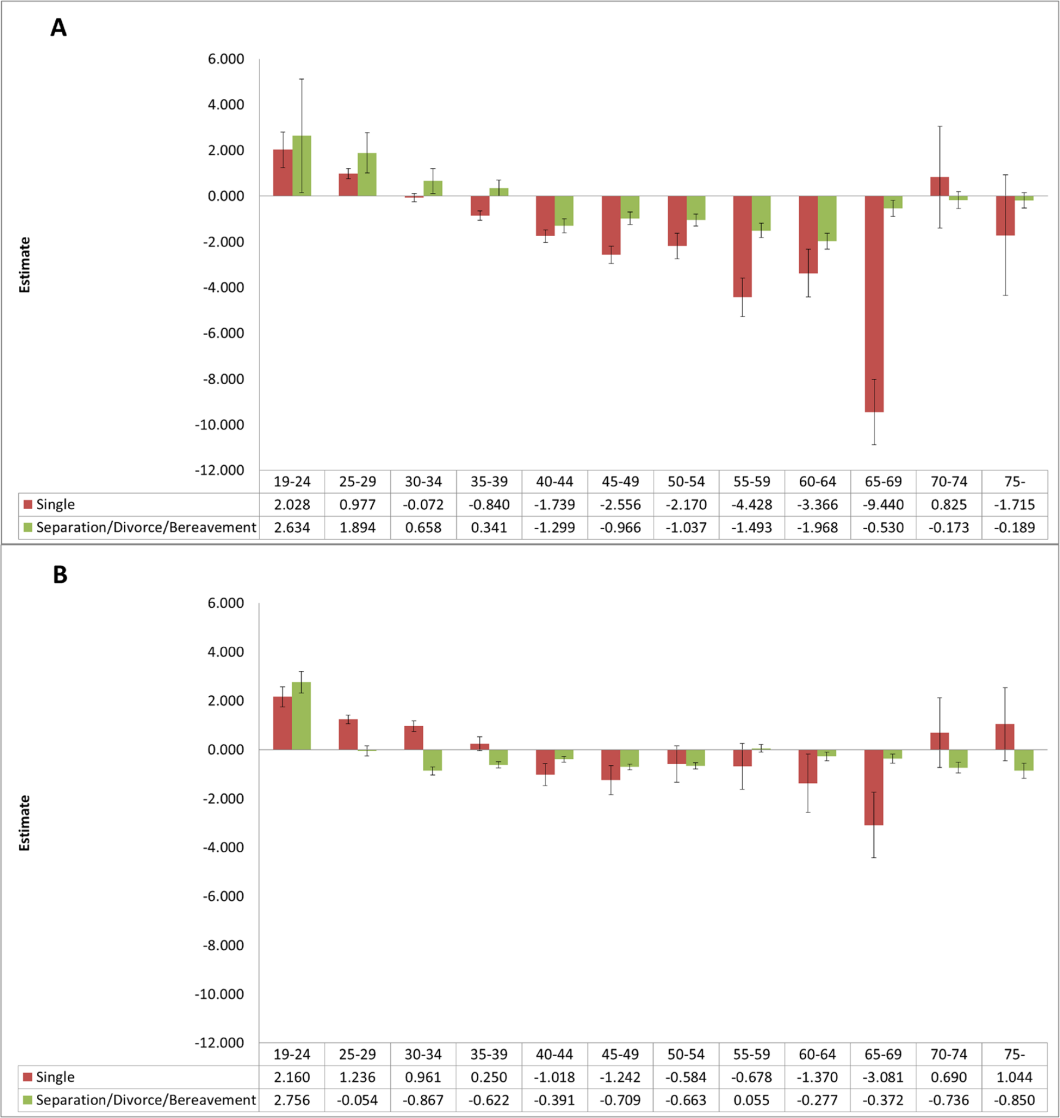
**Regression coefficient estimates for EQ-VAS by marital status and age. A)** Differences in EQ-VAS according to marital status by age group in men, **B)** Differences in EQ-VAS according to marital status by age group in women; *P-value <0.05, *P*-values for results of multilevel analysis. Adjusted for frequent depression for more than 2 weeks, stress awareness, age, family income, education, perceived health status, year of survey, provincial variables. Whiskers in each bar represent standard error estimates of regression coefficients. Reference group is “Married”.

EQ-VAS scores in women under 30 years showed higher QOL scores in the order single > married > marriage problems (age 19–24, single: 2.160, *p* < 0.05; separation/divorce/bereavement: 2.756, *p* < 0.05; age 25–29, single: 1.236, *p* < 0.05; separation/divorce/bereavement: −0.054, *p* > 0.05). In women aged 40–49 years, QOL scores were lower among single than among married women, and between 45 and 54 years, marriage problems were associated with poorer QOL than was married status (Figure 1).

**Figure 2:**
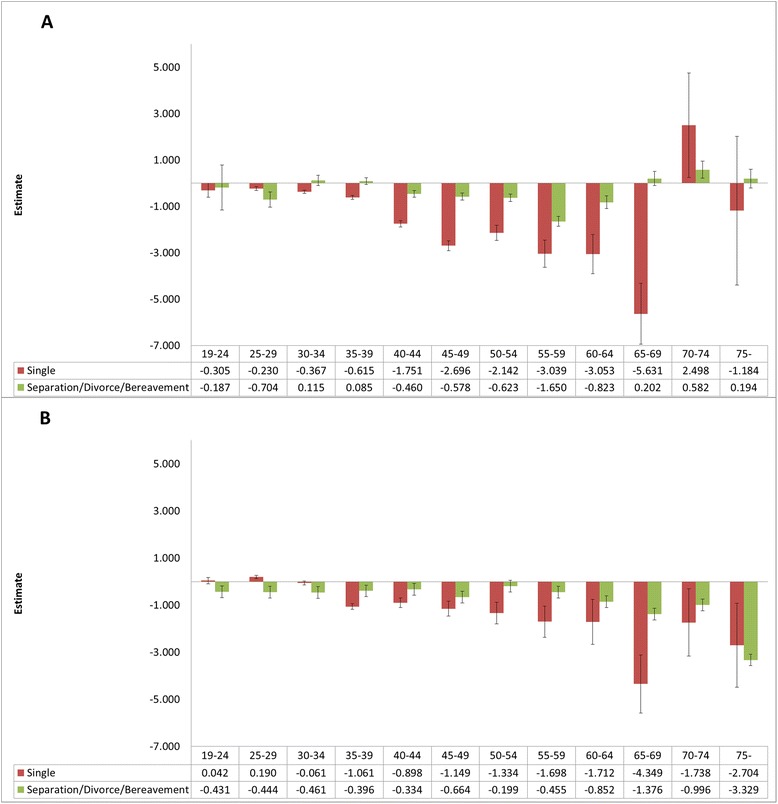
**Regression coefficient estimates for EQ-5D by marital status and age. A)** Differences in EQ-5D according to marital status by age group in men, **B)** Differences in EQ-5D according to marital status by age group in women; *P-value <0.05, *P*-values for results of multilevel analysis. Adjusted for frequent depression for more than 2 weeks, stress awareness, age, family income, education, perceived health status, year of survey, provincial variables. Whiskers in each bar represent standard error estimates of regression coefficients. Reference group is “Married”.

The EQ-5D scores of women under 30 years showed higher QOL scores in the order single > married > marriage problems (age 19–24, single: 0.042, *p* > 0.05; separation/divorce/bereavement: −0.431, *p* > 0.05; age 25–29, single: 0.190, *p* < 0.05; separation/divorce/bereavement: −0.444, *p* < 0.05). Among women over 35 years of age, QOL was higher in the order married > marriage problems > single (Figure 2).

In an additional analysis by dividing the marital status into five groups (single, separation, bereavement, divorced and married), the overall results between marital status and QOL were similar to those when “separation, bereavement and divorced” were collapsed in the analysis. EQ-VAS scores were the lowest in men and women with marital status of separation (single: −0.563, separation: −1.922, and bereavement: −1.152 compared with married in men; single: 0.763, separation:-1.205, bereavement: −0.507 compared with married in women; p < 0.05). EQ-5D scores showed similar trends as EQ-VAS (male single: −0.902, separation: −1.290, bereavement: −0.792; female single: −0.242, separation:-0.980, bereavement: −1.156; p < 0.05). The coefficients for divorced men or women were not statistically significant.

## Discussion

To clarify the causes of low QOL among South Koreans at a national level, targeting adults 19 years of age or older, we focused on marital status as one socioeconomic issue and then analyzed its association with quality of life. Some differences were evident depending on whether we used EQ-5D or EQ-VAS, but we nonetheless observed differences in quality of life depending on marital status in men and women. The EQ-VAS scores of men were a more sensitive indicator of the decline in the quality of life that occurred with marriage problems than the decline associated with single status. However, the decline in QOL associated with single status was greater than that associated with marriage problems using the EQ-5D.

In the case of women, the quality of life of single women was good according to EQ-VAS, and the decline in QOL with marriage problems was high using scores on the EQ-5D. The analysis based on age groups showed that, at younger ages, QOL as measured by EQ-VAS was higher in single men and women. Furthermore, the decrease in QOL due to the marriage problems and being single increased gradually with age. Scores on the EQ-5D for men indicated that QOL was lower among those who had marriage problems and were single regardless of age, but in the case of women, marriage problems and being single were associated with lower QOL in all groups except for women under 30 years of age.

These results show some similarities and differences compared with those of previous studies. According to previous studies, married people have improved mental health compared with those who are single, divorced, or bereaved due to the social relationship with the spouse [13,18–20]. In the present study, married people had higher QOL scores than did those in different marriage status groups generally, but when the results were analyzed by age group, married people under 30 did not have better quality of life than their non-married peers.

Another study examined the relationship of marriage status with mortality and morbidity and found that those who were single, divorced, or bereaved showed higher mortality and morbidity in specific diseases compared with those who were married or cohabiting [21–23]. In the present study, we considered health-related QOL rather than any particular disease, but the positive impact of marriage was similar in both cases. Additionally, previous research on mental health by age group showed that the mental health of single people was better than that of married people in individuals younger than 30 years of age. These results are similar to our study, which showed high QOL among single participants 30–39 years old. However, age groups were not analyzed in detail in the previous study, as they were here, but were divided into three groups. Also, the previous study focused on dermatological patients [10].

This study has some strengths and some limitations. First, the data used were national-level data, making it possible to understand the health of provincial residents, to establish health policies based on evidence, and to evaluate them. Above all, these data reflected the experiences of residents of particular provincialities, not just patients. As also shown in another study, differences in the relationship between marital status and well-being were shared by culture and regions [24], it was meaningful to be able to simultaneously consider the provincial variables in analysis. Furthermore, the study used a large representative sample, and data were collected from a nationwide population. Next, to our knowledge, this is first report on the relationship between QOL (measured by EQ-VAS and EQ-5D) and marital status by age group. Previous studies focused only on QOL by socioeconomic status and the relationship between QOL and marital status in a particular disease. Also, some of those studies did not measure QOL using EQ-VAS or EQ-5D. Finally, this study identified differences in QOL by gender and age group. In some age groups, men had higher QOL scores than women, whereas in other age groups, the reverse was found. This result shows that it is necessary to analyze data for each gender separately when studying quality of life.

However, this study was cross-sectional in nature, hence there is limitation in interpreting the causal relationship between marital status and QOL. But the question about the marital status is for the current time period and the question about the quality of life was on the day the participant filled out the questionnaire. Therefore, we believe that the direction in this relationship is at least from marital status to the quality of life, and it is unlikely that the participants’ previous quality of life resulted in the current marital status. To more accurately measure the relationship between quality of life and marital status, other issues must be considered. For instance, studies are needed about the positive impact of marital satisfaction on quality of life, about marital quality by age group, and about the increase in depression due to marital disruption. Thus, it is important to consider the marital quality in addition to marital status [25–27]. There is also a need to examine the factors leading to the rapid changes in marriage status over time and their impact on the quality of life.

Sudden changes in marital status are expected to have a significant impact on the quality of life in Koreans in the future. Realistically, these changes will be difficult to manage for married and single people. However, it is possible to seek means to help people who are experiencing marriage problems. In particular, intensive management for those in their 40s, the age group with the greatest reduction in quality of life due to marriage problems, should be encouraged.

It is possible for the quality of life to decline due to factors other than marital status, so it is necessary to prevent the decline in QOL in advance through government-level support for people experiencing marriage problems. There is also a need to consider the cultural background of South Korea. Under the influence of Confucianism, Korean society is characterized by a conservative perspective. Thus, bias may occur against people who have experienced a divorce. It is important to develop countermeasures to revise the cultural atmosphere in Korea as part of the solution to the issues faced for those who have experienced marital problems.

## Conclusion

There was a significant relationship between marital status and QOL, and this relationship appeared to differ by gender and age. The results of this study would provide the reference information for developing the management policy for declined QOL.
